# Does Familial Educational Attainment Predict Sleep Quality Trajectories for Older Adults in Mexico?

**DOI:** 10.1093/geroni/igab046.2276

**Published:** 2021-12-17

**Authors:** Connor Sheehan, Margarita Osuna

**Affiliations:** 1 ASU, Tempe, Arizona, United States; 2 USC, University of Southern California, California, United States

## Abstract

Researchers have stressed the importance of sleep for healthy aging and longevity. However, there are few population-level studies of sleep quality focusing on older adults in Latin America and Mexico in particular. The objective of this study is to examine the associations between personal and familial educational attainment on sleep quality. We utilized data from the 2001-2015 Mexican Health and Aging Study (N=4,164; MHAS). Our sample consisted of older adults (aged 50+), married with children. We predicted longitudinal reports of restless sleep across four waves of MHAS using mixed-effects logistic regression. We found that lower levels of respondents’ education, their spouses’ education, and their children’s’ education were associated with lower levels of sleep quality. When the measures of education were included in the same model, one’s own education and children’s education remained significantly associated with quality sleep. Our results stress the importance of familial educational attainment for sleep in Mexico.

